# Games of Skill

**Published:** 1866-08

**Authors:** 


### GAMES OF SKILL.

A CORRESPONDENT asks: "What do you think of GAMES OF SKILL, as *Chess*, *Draughts*, or *Chequers?*" Not understanding such games, our opinion may be of little worth; but we think that human life is too short, its true work too large, and its real object too momentous, to be frittered away with such tom-fooleries. So much for the moral of the subject. As to the mental effects of such employments, they certainly promote habits of deliberation and thoughtfulness, and very important characteristics are they, in this hurry-skurry, helter-skelter, neck-or-nothing age. But far higher purposes would be attained by an equal time spent in the demonstration of some of the problems of Euclid, because they compel the mind to attention, to thoughtfulness, and to habits of legitimate deductions, the want of which is one of the most radical defects of modern education, and one of the most constant causes of making life a failure.

As to the physical tendency of spending hours together, bending over the table, with that insufficient and imperfect breathing which attends an interested mind, any one's common sense will give the answer, that such pastimes are full of mischief, are worse than useless. To all we say, and to invalids and sedentary people especially, when not engaged in the actual and serious business of life, be out and about; sing, whistle, laugh, romp, run, jump, swim, row, ride, do anything, rather than sit still within any four walls, or lounge on a sofa, or doze in a chair, or sleep over a dull book. Moderate and continuous exercise in the open air is without a second, as a means of health, both to the well and to the sick.

---

A LADY SUBSCRIBER, from the sunny South, with forty years' housekeeping experience, says, that the best vinegar is made by allowing a barrel of cider to remain in a cool place for a year or two, and that, after that time, it grows stronger with age. But to a New Yorker, who would have to give a dollar a gallon for real cider, when he can get a gallon of good vinegar for twenty cents, this would be a losing transaction—to say nothing of the "interest on his money:" and not to get that, would kill a Gothamite sooner than the dyspepsia.

